# Is the serum level of survivin, an antiapoptotic protein, a potential predictive and prognostic biomarker in metastatic pancreatic cancer?

**DOI:** 10.1097/MD.0000000000034014

**Published:** 2023-06-23

**Authors:** Nebi Serkan Demirci, Eyyüp Çavdar, Gokmen Umut Erdem, Engin Hatipoglu, Emir Celik, Sevilay Sezer, Ahmet Yolcu, Mutlu Dogan, Erdogan Selcuk Seber

**Affiliations:** a Department of Medical Oncology, Faculty of Medicine, Istanbul University-Cerrahpasa Cerrahpasa, Turkey; b Department of Oncology, Faculty of Medicine, Tekirdag Namik Kemal University, Turkey; c Department of Medical Oncology, Başakşehir Çam and Sakura City Hospital, Turkey; d Department of General Surgery, Faculty of Medicine, Istanbul University-Cerrahpasa Cerrahpasa, Turkey; e Department of Medical Oncology, Haydarpaşa Numune Training and Research Hospital, University of Health Sciences, Turkey; f Department of Biochemistry, Ministry of Health Ankara City Hospital, Turkey; g Department of Radiation Oncology, Tekirdag Namik Kemal University Faculty of Medicine, Turkey; h Department of Medical Oncology, Ankara Oncology Training and Research Hospital, Turkey.

**Keywords:** pancreatic cancer, survival, survivin

## Abstract

In the present study, we aimed to assess the association between the serum survivin level and overall survival and treatment response rates in metastatic pancreatic cancer (MPC). Serum samples were prospectively collected from 41 patients with newly diagnosed MPC patients and 41 healthy individuals (control group) to assess the survivin levels. The median survivin level was 136.2 ng/mL in patients with MPC and 52 ng/mL in healthy individuals (*P* = .028). Patients were divided into low- and high-survivin groups according to the baseline median survivin level. Patients with a high serum survivin level compared with a low serum survivin level had shorter median progression-free survival (2.39 vs 7.06 months; *P* = .008, respectively) and overall survival (3.74 vs 9.52 months; *P* = .026, respectively). Patients with higher serum survivin levels had significantly worse response rates (*P* = .007). The baseline high level of serum survivin in patients with MPC may be associated with treatment resistance and poor prognosis. A confirmation will be needed for these results in future large multicenter prospective studies.

## 1. Introduction

In developed countries, with a 5-year survival rate of 5–8%,^[1,2]^ one of the most deadly cancers and the fourth primary cause of cancer death is pancreatic cancer. Despite many treatment modalities, the median survival duration in metastatic disease remains <12 months.^[3]^ At the time of diagnosis, only 10–15% of patients are in the operable stage and the effective treatment method is still surgery. Therefore, early diagnosis improves survival by increasing the effectiveness of surgery.^[4,5]^ Radiological techniques and serological markers are used for diagnosis, and many biomarkers in the serum and plasma have been identified. Several blood biomarkers, such as carbohydrate antigen 19-9 (CA 19-9), carcinoembryonic antigen (CEA), lactate dehydrogenase, caveolin-1, C-reactive protein, and cathepsin L, have prognostic importance in pancreatic cancer.^[6–9]^

These markers are inexpensive and simple to evaluate in blood samples. However, due to the poor prognosis of patients with pancreatic cancer, novel biomarkers are still required to determine the prognosis of the disease and predict the response to treatment.

The inhibition of apoptosis is very important in cancer development. This inhibition not only accelerates tumor development but also leads to the development of resistance to treatment.^[10–13]^ Survivin is known as the tiniest member of the inhibitor of the apoptosis protein family. The survivin gene on chromosome 17q25 consists of 3 introns and 4 exons and encodes a 142-amino acid protein. In addition to antiapoptotic activity, this protein plays a role in cell proliferation and angiogenesis.^[14–16]^ It is expressed in the G2/M phase of the cell cycle at the highest rate to support cell division, whereas its level in the G1 phase is lower.^[17]^ Survivin is detected at insignificant levels in most normal adult tissues, with the exception of the testis, thymus, and placenta; however, it is commonly expressed in human tissues during fetal development.^[16,18]^ Inhibiting apoptosis and increasing cell proliferation, survivin advances tumor growth and progression.^[13,19]^

Survivin expression is associated with poor survival in most cancers. Previous studies have reported that survivin is prognostic for a variety of cancers such as those of the lung, prostate, breast, colon, and pancreas. It has been shown that higher survivin expression is also associated with higher recurrence rates and treatment resistance.^[20–26]^ It has been shown that healthy individuals having a high level of survivin were associated with a greater risk of developing cancer. From this point of view, survivin levels may also have diagnostic value. It has been shown that healthy individuals having a high level of survivin were associated with a greater risk of developing cancer. From this point of view, survivin levels may also have diagnostic value_._^[27–30]^

Following determining the serum levels of basal survivin in patients with metastatic pancreatic cancer (MPC), we analyzed the correlations between this level and the clinicopathological features of the patients in our study. We also investigated the relationships between the survivin level with prognosis and treatment response.

## 2. Patients and methods

### 2.1. Study population

This prospective cross-sectional controlled study included 41 patients and 41 healthy controls between March 2018 and April 2020. Eligible patients were aged at least 18 years and had histologically or cytologically confirmed pancreatic ductal adenocarcinoma; distant metastatic disease; an Eastern Cooperative Oncology Group performance status rating of 2 or less; and adequate hematological, renal, hepatic, and cardiac function. Each patient was metastatic at the time of diagnosis and had not received any previous treatment. Patients with central nervous system metastases, second primary malignancies, active infections, or those who were pregnant or breastfeeding were excluded from this study. For assessing survivin and other tumor markers (CEA, CA19-9, and lactate dehydrogenase), we obtained serum samples of patients with cancer at baseline. Complete blood cell count, serum electrolyte, serum creatinine, and liver function tests at baseline were analyzed in all patients. Furthermore, serum samples of healthy individuals were collected to assess their survivin levels. A detailed history, physical examinations, blood tests, tumor marker tests (CA19-9, CEA), and abdominal sonography of healthy individuals were assessed. Participants with no evidence of pancreatic disease including diabetes mellitus, chronic pancreatitis, or other abnormalities were enrolled as healthy individuals.

Chemotherapy based on the gemcitabine chemotherapy regimen (intravenous infusion of 1200 mg/m^2^ gemcitabine on days 1 and 8, every 21 days for a maximum of 6 cycles) as the first-line treatment was given to each patient. Patients received chemotherapy unless disease progression or unacceptable toxicity was experienced. We enrolled all patients who had completed at least 1 cycle of therapy in the study.

Tumor response was assessed using Response Evaluation Criteria in Solid Tumors (version 1.1) through computed tomography or magnetic resonance imaging at baseline and after chemotherapy.

The Institutional Ethics Committee approved the study and all participants provided written informed consent.

### 2.2. Analysis of serum survivin

We collected all blood samples through venipuncture from the antecubital vein, between 8:00 am and 9:00 am following overnight fasting of 8–10 hours before and after 3 cycles of chemotherapy. The plasma was divided through centrifugation at 2500 rpm for 20 minutes at room temperature, within 30 minutes of blood collection. A human survivin enzyme-linked immunosorbent assay (ELISA) kit (Elabscience Biotechnology Co., Ltd, Houston, TX; PRC; Catalog No: E-EL-H1584) was used for the quantitative determination of survivin levels in serum. The kit protocol is based on the Sandwich-ELISA method. The micro-ELISA plate supplied in this kit is precoated with an antibody particular to survivin. We added standards or samples to suitable micro-ELISA plate wells and united them with the specific antibody. A biotinylated detection antibody particular for the human survivin and avidin-horseradish peroxidase conjugate was annexed to each microplate well afterward, and the plate was incubated. Free components were washed out, and substrate solution was annexed. After a short incubation period, the enzyme reaction was stopped, and the color generated in each well was read at 450 nm using an automated ELISA reader (BioTek, La Puente, Los Angeles, CA; Elx800 Chemistry Analyzer). The quantified optical intensity is straightforwardly proportional to the concentration of survivin in both the standards and samples.

### 2.3. Statistical analysis

For all statistical analyses, we used Statistical Package for Social Sciences software version 22.0 for Windows (SPSS, Inc, Chicago, IL). Descriptive statistics were reported as percentage and median. The association of clinicopathological variables and expression levels of survivin were analyzed using the chi-squared test or Fisher exact test. A value of *P* < .05 was considered statistically significant. The differences in the serum survivin concentration between cancer patients and the control group were carried out by Mann–Whitney *U*-test.

The Kaplan–Meier method was used to analyze the survival data. Univariate analysis was used to estimate hazard ratios (HRs) and their 95% confidence intervals (CIs) for overall survival (OS) and progression-free survival (PFS). Variables associated with *P* < .1 or clinically significant variables in the univariate Cox regression analysis were subjected to multivariate analysis using backward stepwise selection. OS was defined as the duration from the date of diagnosis to death or last follow-up, and PFS was defined as the time from the initiation of first-line chemotherapy to disease progression or death from any cause. Patients who were lost to follow-up were not included.

## 3. Results

A total of 41 patients with pancreatic cancer and 41 healthy individuals were recruited in this study. Table 1 shows the baseline characteristics of the patients. The median follow-up period of time was 5.7 months (range, 0.43–35.9 months) after the first diagnosis. The median age was 59 years (range, 44–80 years) for MPC patients and 58 years (range, 28–79 years) for healthy control subjects. The male-to-female ratio was 3.1 (31/10) and 1.27 (23/18) for patients and healthy subjects, respectively. CA19-9, CEA, and the basal levels of survivin were analyzed in the plasma samples taken from the patients. The cutoff value of CEA and CA19-9 levels were 5 ng/mL and 37 U/mL, respectively. 53.7% and 90.2% of the patients had high CEA and CA19-9 levels, respectively. The median number of chemotherapy cycles was 3 (range, 1–6). Moreover, following the first 3 cycles of chemotherapy, 44% (n = 18) of the patients could not be examined due to either death or loss of follow-up. The rest of the patients 56% (n = 23) were examined after 3 cycles.

**Table 1:** Baseline patient demographics and clinical characteristics.

Characteristics	No. of patients, N = 41 (%)
Age, years	
Median	59
Range	44-80
<65	31 (75.6)
≥65	10 (24.4)
Gender	
Male	31 (75.6)
Female	10 (24.4)
Smoking	
Yes	27 (65.9)
No	14 (34.1)
Alcohol use	
Yes	7 (17.1)
No	34 (82.9)
Comorbidity	
Yes	27 (65.9)
No	14 (34.1)
Tumor localization	
Head	24 (58.5)
Body and/or tail	17 (41.5)
Baseline survivin	
High	21 (51.2)
Low	20 (48.8)
Baseline CEA	
Normal	19 (46.3)
High	22 (53.7)
Baseline CA 19-9	
Normal	4 (9.8)
High	37 (90.2)

CA 19-9 = carbohydrate antigen 19-9, CEA = carcinoembryonic antigen.

Range: minimum–maximum.

The median survivin level was 136.2 ng/mL (4.7–5004 ng/mL; mean, 787.68 ± 1420.87 ng/mL) for the patients and 52 ng/mL (3.8–5033 ng/mL; mean, 707.35 ± 1400.12 ng/mL) in the control group. The patients were further categorized into low- and high-survivin groups based on the median baseline survivin level. No significant relationships were observed between the baseline serum survivin level and other clinical characteristics (Table 2). The difference in survivin level between the healthy and patient groups was statistically significant (*P* = .028).

**Table 2:** Clinicopathological features in relation to the baseline survivin levels.

	Survivin levels		
Features	<136 (n = 20)	≥136 (n = 21)	*P* value
Age, years (<65/≥65)	15/5	16/5	.484
Gender (m/f)	14/6	17/4	.370
Smoking (yes/no)	12/8	15/6	.440
Alcohol use (yes/no)	3/17	4/17	1
Comorbidity (yes/no)	13/7	14/7	.910
Tumor localization (head/body or tail)	11/9	13/8	.654
Baseline CEA (normal/high)	10/10	9/12	.647
Baseline CA 19-9 (normal/high)	1/19	3/18	.606
Response to therapy (yes/no)	12/8	4/17	.007

CA 19-9 = carbohydrate antigen 19-9, CEA = carcinoembryonic antigen, f = female, m = male.

The clinical response to chemotherapy was divided into 2 groups progressive (progressive disease) or nonprogressive (partial response + stable disease). Patients with higher serum survivin levels had significantly worse response rates (*P* = .007) (Table 2).

**Figure 1.**
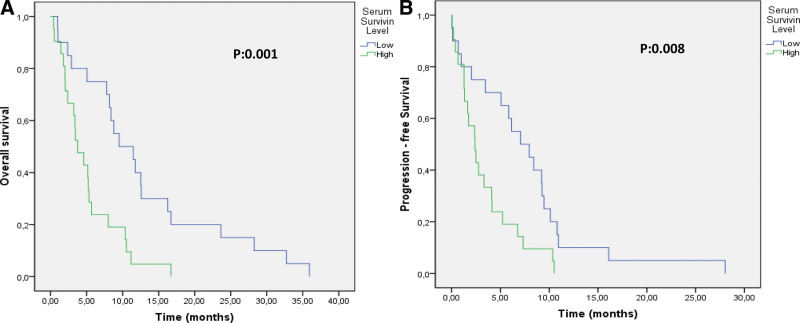
Kaplan–Meier plots illustrate the shorter time to (A) overall and (B) progression-free survival in the high survivin group compared with the low survivin group.

From univariate analysis, the median PFS was 7.06 months in patients with low serum survivin levels, whereas it was 2.39 months in patients with high serum survivin levels (*P* = .008) (Fig. 1B and Table 3). In addition, from univariate analysis, PFS differed significantly according to sex, smoking status, treatment response, and tumor localization. Based on multivariate analysis, sex, treatment response, and tumor localization were shown to be related to PFS. Nevertheless, no significant relationship between PFS and the baseline survivin level (Table 3) was not found.

**Table 3:** Univariate and multivariate survival analysis according to clinicopathological features.

		Progression-free survival (PFS)				Overall survival (OS)			
		Univariate analysis		Multivariate analysis		Univariate analysis		Multivaraite analysis	
Characteristics	No. of patients, N = 41 (%)	mPFS (mo)	*P*	HR (95% CI)	*P*	mOS (mo)	*P*	HR (95 % CI)	*P*
Age, years									
Median	59								
Range	44–80								
<65	31 (75.6)	3.45				7.78			
≥65	10 (24.4)	4.14	.409			5.15	.655		
Gender									
Male	31 (75.6)	5.06				5.32			
Female	10 (24.4)	0.98	.015	2.710 (1.11–6.6)	.028	5.68	.487		
Smoking									
Yes	27 (65.9)	5.22				7.78			
No	14 (34.1)	1.31	.003	1.615 (0.483–5.397)	.436	3.74	.243		
Alcohol use									
Yes	7 (17.1)	5.06				5.32			
No	34 (82.9)	2.76	.714			5.68	.479		
Comorbidity									
Yes	27 (65.9)	3.31				5.22			
No	14 (34.1)	4.1	.815			5.68	.478		
Tumor localization									
Head	24 (58.5)	5.84				8.18			
Body and/or tail	17 (41.5)	1.31	.022	2.840 (1.36–5.89)	.005	3.38	.316		
Baseline survivin									
High	21 (51.2)	2.39				3.74			
Low	20 (48.8)	7.06	.008	2.353 (0.937–5.905)	.068	9.52	.001	2.284 (1.09–4.77)	.026
Baseline CEA									
Normal	19 (46.3)	5.84				8.18			
High	22 (53.7)	2.76	.475			5.32	.6		
Baseline CA 19-9									
Normal	4 (9.8)	2.76				3.74			
High	37 (90.2)	4.1	.967			5.68	.56		
Treatment response									
Yes	16 (9.8)	9.23				11.76			
No	25 (90.2)	1.74	≤.001	4.470 (1.80–11.08)	.001	3.38	≤.001	4.277 (2–9.14)	≤.001

CA 19-9 = carbohydrate antigen 19-9, CEA = carcinoembryonic antigen, CI = confidence interval, HR = hazard ratio, PFS = progression-free survival.

The median OS was 5.6 months. A high serum survivin level was associated with poor survival. The median OS was 9.52 months in patients with low survivin levels, whereas it was 3.74 months in patients with high survivin levels (HR, 2.284; 95% CI, 1.09–4.77; *P* = .026) (Fig. 1A and Table 3). In addition, the OS of patients responding to treatment was significantly better based on both univariate and multivariate analysis (HR, 4.277; 95% CI, 2–9.14; *P* < .001) (Table 3).

## 4. Discussion

The study aimed to find out whether the serum survivin level is predictive of treatment response and survival. Apoptosis is an important cellular activity in tumorigenesis that has been widely reported to be inhibited in many types of cancer, such as breast, liver, stomach, pancreatic, and lung cancers.^[16,25,31–33]^ Two apoptotic pathways exist: the intrinsic and extrinsic pathways. Both are driven by a good deal of caspase proteins, such as initiator caspases (caspase-8 and -9) and effector caspases (caspase-3, -6, and -7). Caspase-3 and -7 are activated following the activation of caspase-9.^[25,33–36]^ As an apoptosis inhibitor, survivin has become an interesting potential biomarker for cancer in recent years. Increased survivin levels are associated with the inhibition of cell death induced by extrinsic or intrinsic apoptotic means.^[37,38]^ The cell cycle regulates survivin expression. In addition, survivin can be phosphorylated by CDK1, an essential kinase for cell cycle regulation.^[39]^ Survivin is also directly associated with the Wnt/β-catenin and Notch signaling pathways. Additionally, it interacts with the STAT3 oncogene and P53 tumor suppressor gene.^[40,41]^ For that reason, survivin has a significant role in carcinogenesis.

Several studies have shown that there is a relationship between pancreatic cancer and the overexpression of survivin in tumor tissues^[37,42–45]^; however, few studies have investigated the association between the serum survivin level and prognosis in pancreatic cancer.^[26,42,43,46–48]^ Previous studies investigating serum survivin levels have focused mostly on comparing the survivin level between patients and healthy individuals, similar to our report. We showed that our patients had higher serum survivin levels compared to the healthy controls. Additionally, we demonstrated that these high levels were associated with poor survival. However, no significant differences in the serum survivin level were found among MPC patients with different ages, sexes, tumor localization, smoking status, alcohol use, comorbid conditions, and baseline CEA and CA19-9 levels. Previous studies have shown that clinicopathological parameters and survivin levels are directly related, whereas other studies have produced contradictory results.^[49–51]^ In patients with pancreatic cancer, the serum survivin level is not known to be correlated with age, sex, or tumor size.^[43]^

The baseline survivin level could be used to predict the chemotherapy response in patients with MPC.^[13,45,49–52]^ In our study, we found that patients with low low baseline serum survivin levels had better chemotherapy responses. Conversely, in another study evaluating patients with lung cancer with higher tissue survivin expression, a better response was demonstrated after chemotherapy.^[49]^ It remains unclear how survivin plays a role in the response to treatment. Current studies indicate that survivin may regulate DNA repair by interacting with DNA repair components. Breast cancer patients with high survivin levels tended to exhibit good responses to chemotherapy while responding poorly to endocrine therapy. On the other hand, low serum survivin levels have been shown to be associated with a slightly better response in patients with pancreatic cancer.^[53]^ These findings may increase our understanding of the different functions of the survivin protein.^[54–56]^ We found that only the baseline serum survivin level was associated with the therapeutic response among the clinicopathological features. Nevertheless, this may be related to the small sample size.

Low survivin expression in tumor tissue has been associated with better survival rates in various types of cancers, including bladder, esophageal, pancreatic, lung, colorectal, breast, gastric, ovarian, and gallbladder cancers as well as medulloblastomas and gliomas.^[23,24,43,46,49,57–65]^ By contrast, some studies have shown that patients with high survivin levels exhibit improved survival. Indeed, based on these previous reports, survivin tissue levels may be prognostic for patients with pancreatic cancer.^[43,58,63]^ In addition to these survival data on tissue expression levels of survivin, serum survivin levels have also been associated with poor survival in patients with pancreatic cancer.^[43]^ We found that OS was significantly shorter in patients with high baseline serum survivin levels. Besides, univariate analysis in the present study revealed the prognostic significance of clinicopathological factors such as sex and smoking status. In the multivariate analysis, we evaluated the prognostic and predictive value of the serum survivin level and identified survivin as an independent prognostic and predictive factor.

As expected, the PFS was directly associated with the response to chemotherapy. Based on univariate analysis, the PFS rate was significantly lower in patients with high serum survivin levels, whereas in the multivariate analysis, this difference was not significant. This may be due to the fact that the study sample comprised a small, non-normally distributed patient group.

Studies on the use of serum survivin levels as a diagnostic marker have shown that patients with hepatocellular carcinoma and acute lymphoblastic leukemia have higher serum survivin levels than healthy individuals.^[27–30]^ However, in contrast to these studies, some studies in patients with lung cancer did not show a significant increase in serum survivin levels.^[28,66]^ Since the aim of our study was to examine the relationship between survivin levels, survival, and response to treatment in patients with MPC, survivin was not evaluated as an early diagnosis marker. Healthy individuals whose blood samples were taken were not followed up.

Limitations of this study include that it is a single-center study involving a limited number of patients. In addition, there was a lack of information about the correlation between serum survivin and the expression of survivin in tumor tissues. Another limitation was that serum survivin levels were not measured after chemotherapy. Nevertheless, our study has many strengths. To the best of our knowledge, this is the largest study evaluating correlations between the baseline survivin level and the prognosis and treatment response in patients with MPC. The treatment was homogeneous because all patients underwent the gemcitabine regimen. In addition, the serum survivin levels of patients with pancreatic cancer were compared with those of a healthy control group.

## 5. Conclusion

The chemotherapy response and OS in patients with MPC may be better by low baseline serum survivin levels. Nevertheless, we will need larger prospective studies to confirm these results.

## Author contributions

**Conceptualization:** Nebi Serkan Demirci, Eyyüp Çavdar, Ahmet Yolcu, Erdogan Selcuk Seber.

**Data curation:** Nebi Serkan Demirci, Eyyüp Çavdar, Gokmen Umut Erdem, Sevilay Sezer, Ahmet Yolcu, Mutlu Dogan, Erdogan Selcuk Seber.

**Formal analysis:** Nebi Serkan Demirci, Eyyüp Çavdar, Gokmen Umut Erdem, Engin Hatipoglu, Emir Celik, Ahmet Yolcu, Mutlu Dogan, Erdogan Selcuk Seber.

**Funding acquisition:** Nebi Serkan Demirci, Eyyüp Çavdar, Ahmet Yolcu, Erdogan Selcuk Seber.

**Investigation:** Nebi Serkan Demirci, Eyyüp Çavdar, Ahmet Yolcu, Erdogan Selcuk Seber.

**Methodology:** Nebi Serkan Demirci, Eyyüp Çavdar, Gokmen Umut Erdem, Ahmet Yolcu, Erdogan Selcuk Seber.

**Project administration:** Nebi Serkan Demirci, Eyyüp Çavdar, Emir Celik, Sevilay Sezer, Ahmet Yolcu, Erdogan Selcuk Seber.

**Resources:** Nebi Serkan Demirci, Eyyüp Çavdar, Ahmet Yolcu, Erdogan Selcuk Seber.

**Software:** Nebi Serkan Demirci, Eyyüp Çavdar, Gokmen Umut Erdem, Engin Hatipoglu, Emir Celik, Sevilay Sezer, Ahmet Yolcu, Mutlu Dogan, Erdogan Selcuk Seber.

**Supervision:** Nebi Serkan Demirci, Eyyüp Çavdar, Gokmen Umut Erdem, Engin Hatipoglu, Emir Celik, Ahmet Yolcu, Mutlu Dogan, Erdogan Selcuk Seber.

**Validation:** Nebi Serkan Demirci, Eyyüp Çavdar, Gokmen Umut Erdem, Engin Hatipoglu, Emir Celik, Sevilay Sezer, Ahmet Yolcu, Mutlu Dogan, Erdogan Selcuk Seber.

**Visualization:** Nebi Serkan Demirci, Eyyüp Çavdar, Gokmen Umut Erdem, Engin Hatipoglu, Emir Celik, Sevilay Sezer, Ahmet Yolcu, Mutlu Dogan, Erdogan Selcuk Seber.

**Writing – original draft:** Nebi Serkan Demirci, Eyyüp Çavdar, Gokmen Umut Erdem, Engin Hatipoglu, Emir Celik, Sevilay Sezer, Ahmet Yolcu, Mutlu Dogan, Erdogan Selcuk Seber.

**Writing – review & editing:** Nebi Serkan Demirci, Eyyüp Çavdar, Gokmen Umut Erdem, Engin Hatipoglu, Emir Celik, Sevilay Sezer, Ahmet Yolcu, Mutlu Dogan, Erdogan Selcuk Seber.
